# Factors Predicting Effectiveness of Eradication Therapy for *Helicobacter pylori*-Associated Dyspepsia Symptoms

**DOI:** 10.3390/life14080935

**Published:** 2024-07-25

**Authors:** Kohei Yasuda, Daisuke Chinda, Tadashi Shimoyama, Tetsu Arai, Kazuki Akitaya, Sae Fujiwara, Hiroki Nomiya, Yoshio Sasaki, Kazuo Komai, Yoshihiko Sawada, Yoshiharu Saito, Hironobu Chiba, Hirotake Sakuraba, Shinsaku Fukuda, the RINGO Study Group

**Affiliations:** 1 Department of Gastroenterology and Hematology, Hirosaki University Graduate School of Medicine, Hirosaki 036-8562, Japan; y9105883314@yahoo.co.jp (K.Y.); teddyscello@icloud.com (T.A.); kazukiakitaya0926@yahoo.co.jp (K.A.); saekudo0803@gmail.com (S.F.); h.nomiya@hirosaki-u.ac.jp (H.N.); hirotake@hirosaki-u.ac.jp (H.S.); sfukuda@hirosaki-u.ac.jp (S.F.); 2 Division of Endoscopy, Hirosaki University Hospital, Hirosaki 036-8563, Japan; 3 Department of Internal Medicine, Aomori General Health Examination Center, Aomori 030-0962, Japan; tsimo@hirosaki-u.ac.jp; 4 Sasaki Clinic of Gastroenterology and Internal Medicine, Aomori 030-0914, Japan; yossy_pleasure_19690920@nifty.com; 5 Komai Clinic of Gastroenterology and Internal Medicine, Aomori 030-0947, Japan; mrshmdt4144@mg.point.ne.jp; 6 Sawada Clinic of Internal Medicine, Hirosaki 036-8261, Japan; ysawada@jomon.ne.jp; 7 Shinjo Clinic of Gastroenterology and Internal Medicine, Aomori 038-0042, Japan; yosiharu@jomon.ne.jp; 8 Chiba Clinic of Gastroenterology and Internal Medicine, Hirosaki 036-8316, Japan; chibatti2002@gmail.com; 9 Risk Investigation of Gastric Cancer and Observation after Eradication Study Group, Hirosaki 036-8562, Japan

**Keywords:** functional gastroduodenal disorder, functional dyspepsia, *Helicobacter pylori*-associated dyspepsia, *H. pylori* eradication, serum pepsinogen, smoking habit, modified Frequency Scale for the Symptoms of Gastroesophageal Reflux Disease (FSSG)

## Abstract

Functional dyspepsia is distinguishable from *Helicobacter pylori*-associated dyspepsia. However, distinguishing *H. pylori*-associated dyspepsia from functional dyspepsia before *H. pylori* eradication is difficult. Therefore, in the present study, we aimed to investigate whether serum pepsinogen levels before *H. pylori* eradication are associated with the amelioration of dyspepsia after successful *H. pylori* eradication. Additionally, we examined the usefulness of serum pepsinogen levels and other factors in predicting dyspepsia outcomes. *H. pylori* eradication was effective in 14 patients (Responders) and ineffective in 19 patients (Non-responders). The pepsinogen I/II ratio in Responders (3.4 ± 1.2) and Non-responders (2.3 ± 1.0) differed significantly (*p* = 0.006). The optimal cut-off pepsinogen I/II value was 2.3. Multivariate logistic regression analysis showed that the adjusted odds ratio for Non-responders was 26.1 (95% confidence interval: 2.0–338.0, *p* = 0.012) for a pepsinogen I/II ratio ≤ 2.3 and 8.10 (95% confidence interval: 1.1–57.6, *p* = 0.037) for smoking habits. The pepsinogen I/II ratio and smoking habits were associated with the effects of *H. pylori* eradication on dyspeptic symptoms. Thus, the pepsinogen I/II ratio cut-off value can be used to identify patients likely to respond to *H. pylori* eradication after the resolution of dyspeptic symptoms.

## 1. Introduction

Functional gastroduodenal disorders are classified into four categories: functional dyspepsia (FD) (postprandial distress syndrome and epigastric pain syndrome), belching disorders (excessive gastric and supragastric belching), chronic nausea and vomiting disorders (chronic nausea vomiting syndrome, cyclic vomiting syndrome, and cannabinoid hyperemesis syndrome), and rumination syndrome. FD is characterized by one or more of the following symptoms: postprandial fullness, early satiation, epigastric pain, and epigastric burning that are unexplained after a routine clinical evaluation. In the Rome IV diagnostic criteria established in 2016, FD is defined as symptoms that are bothersome and troublesome, persist for at least 2 months, occur on at least 4 days per month, and cannot be fully explained by other diseases even after appropriate evaluation [1]. In Japan, the prevalence of FD is 11–17% among health check-up recipients [2,3]. Dyspeptic symptoms are caused by multiple factors, including gastrointestinal dysmotility, visceral hypersensitivity, gastric acid secretion, and lifestyle choices such as exercise, sleep, and diet [4,5]. Therefore, patients with FD are often advised to make dietary modifications such as avoiding spicy or fatty foods, reducing caffeine and alcohol intake, and consuming smaller, more frequent meals. As for pharmacological treatment, proton pump inhibitors (PPIs) are commonly used to reduce stomach acid production and may provide relief in some patients with FD. Prokinetics can enhance stomach emptying and alleviate symptoms such as early satiation. Commonly prescribed prokinetics include acotiamide, metoclopramide, and domperidone. In addition, tricyclic antidepressants can be prescribed in low doses to help manage the pain and discomfort associated with FD by affecting the perception of pain in the brain and exerting some prokinetic effects [5,6,7,8,9].

*Helicobacter pylori* is a gram-negative bacterium with a helical shape that chronically infects the human gastric epithelium [10,11,12]. *H. pylori* eradication requires pharmacological treatment with appropriate antibiotic regimens, which are recommended for all patients who are positive for *H. pylori* [13]. *H. pylori* infection can induce dyspeptic symptoms. Malfertheiner et al. investigated whether *H. pylori* eradication leads to long-term relief of symptoms in FD. Among 800 patients who tested positive for *H. pylori* and had dyspeptic symptoms, the *H. pylori* eradication group showed significant amelioration of symptoms compared with the group administered lansoprazole alone. This finding suggests that *H. pylori* infection causes dyspeptic symptoms in certain patients with FD, who may experience long-term symptom amelioration after *H. pylori* eradication [14]. In a meta-analysis, *H. pylori* eradication significantly ameliorated dyspeptic symptoms, with a relative risk of 0.91 (95% confidence interval [CI]: 0.87–0.94). Overall, these studies suggest that the number needed for treatment (NNT) is 13 (95% CI: 9–19) [15].

Thus, if dyspeptic symptoms are relieved after *H. pylori* eradication, patients are considered to show *H. pylori*-associated dyspepsia. In contrast, if symptoms are not alleviated, FD can be distinguished from *H pylori*-associated dyspepsia [16]. Dyspeptic symptoms are caused by several mechanisms related to gastric acid and gastric emptying. As gastric mucosal atrophy progresses in the stomach corpus, gastric acid secretion is reduced, and gastric emptying is delayed [17]. *H. pylori* eradication alleviates gastric mucosal inflammation and increases acid secretion, including mucosal atrophy restoration [18]. These changes may be associated with dyspeptic symptom amelioration. The most convenient method to assess gastric mucosal inflammation and atrophy is measuring serum pepsinogen (PG) levels [19,20,21]. Serum PG I levels are positively correlated with gastric acid secretion [22]. Additionally, gastric acid secretion and mucosal inflammation are affected by several factors. For example, body mass index (BMI) is positively correlated with gastric acid secretion, whereas smoking promotes atrophic gastritis and intestinal metaplasia in patients infected with *H. pylori* [23].

*H. pylori*-associated dyspepsia is included in the Rome IV criteria and has been recognized worldwide [1]. Remission of symptoms for over 6 months after successful eradication distinguishes dyspepsia from FD. However, patients should not have to experience dyspeptic symptoms for over 6 months; therefore, *H. pylori*-associated dyspepsia should be predicted before eradication. If *H. pylori*-associated dyspepsia is predicted before eradication, many patients may experience early alleviation of symptoms. Therefore, in the present study, we aimed to investigate whether serum PG levels before *H. pylori* eradication are associated with the alleviation of dyspeptic symptoms after successful *H. pylori* eradication. Additionally, we evaluated the cut-off value of serum PG levels and other factors to identify patients in whom dyspeptic symptoms would persist after successful eradication.

## 2. Materials and Methods

### 2.1. Patients

**Figure 1:**
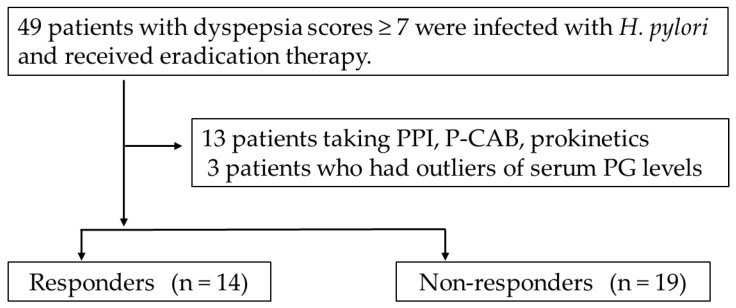
Flowchart of the study design. Abbreviations: PPIs, proton pump inhibitors; P-CABs, potassium-competitive acid blockers.

Patients who attended the Risk Investigation of Gastric Cancer and Observation after the eradication (RINGO) study between July 2013 and July 2014 were enrolled in the study. We excluded patients taking PPIs, potassium-competitive acid blockers, and prokinetics; those who had serum PG I and PG II outliers (outliers are defined as data that are equal to or greater than the third quartile +3.0 times the interquartile range); and those whose data could not be followed (Figure 1, Tables S1 and S2). Patient height and body weight were recorded, and dyspeptic symptoms were evaluated by having patients complete a modified Frequency Scale for the Symptoms of Gastroesophageal Reflux Disease (FSSG) questionnaire [24].

The FSSG questionnaire is the standard questionnaire used for diagnosis of gastroesophageal reflux disease (GERD) and assessment of response to treatment [25]. Developed by Kusano and colleagues, the FSSG questionnaire originally consisted of a total of 12 questions, comprising seven questions related to acid reflux symptoms and 5 questions related to dyspeptic symptoms. Subsequently, Kusano and colleagues modified the questionnaire by adding two questions regarding interdigestive and postprandial epigastric pain so that it was also diagnostic for FD [24]. The modified FSSG questionnaire consists of a total of 14 questions that can be categorized into two groups: seven questions pertain to acid reflux symptoms and the remaining seven relate to dyspeptic symptoms. The 14 questions are given in Table S3. We referred to seven questions (#1, 4, 6, 7, 9, 10, and 12) as the reflux symptom questions, and these were used to calculate the reflux score. The other seven questions (#2, 3, 5, 8, 11, 13, and 14) were used to calculate the dyspepsia score. The cut-off value of dyspepsia scores for diagnosing FD was ≥7 points, with a sensitivity of 0.76 and a specificity of 0.83 [26]. In this study, the modified FSSG questionnaire was used, and dyspepsia scores ≥ 7 were considered to indicate dyspeptic symptoms.

We asked the patients about their smoking habits, alcohol consumption, and underlying diseases. Smoking habits were compared between past or current smokers (defined as patients who smoked or were currently smoking at least one cigarette per day, respectively) and nonsmokers. Alcohol consumption was compared between past or current drinkers and non-drinkers. Eighteen of the 33 patients had underlying diseases (including hypertension, hyperlipidemia, asthma, gout, diabetes mellitus Basedow’s disease, iron-deficiency anemia, allergic rhinitis, cerebral infarction, insomnia, and arrhythmia). These details were collected via questionnaires.

### 2.2. Diagnosis of H. pylori Infection and Serum PG Levels

*H. pylori* infection was diagnosed by measuring serum anti-*H. pylori* IgG antibody levels and performing a urea breath test (UBT) and/or stool antigen test (SAT). An enzyme immunoassay (EIA) kit, E-plate (Eiken Chemical Co. Ltd., Tokyo, Japan), was used to test the serum anti-*H. pylori* IgG antibodies, with an antibody titer ≥10 U/mL and <3 U/mL indicating positive and negative result, respectively. UBT was performed using a UBiT tablet (Otsuka Pharmaceutical Co., Ltd., Tokyo, Japan) containing 100 mg of ^13^C-urea. The UBT result was considered positive when the increase in the Δ^13^C value at 20 min after ^13^C-urea administration was greater than 2.5‰ [27]. *H. pylori* antigen in stool samples was measured using Testmate Pylori Antigen EIA (Wakamoto Co., Ltd., Tokyo, Japan; Minaris Medical Co., Ltd., Tokyo, Japan) [28]. Patients who showed positive results for serum antibodies, UBT, and/or SAT were diagnosed with *H. pylori* infection. Serum PG I and PG II levels were measured using latex immunoassay; the PG I/II ratios were calculated using these values.

### 2.3. Evaluation of H. pylori Eradication Efficacy

All patients underwent upper gastrointestinal endoscopy to confirm that they had no organic disease to explain their dyspeptic symptoms, excluding *H. pylori* infection-related gastritis. *H. pylori* eradication therapy was administered, and successful eradication was confirmed using UBT or stool antigen testing after at least 4 weeks. Thirty-three patients were assessed for symptoms using the modified FSSG questionnaire after successful eradication. Among those approved by the Japanese health insurance system, first-line eradication consisted of 1 week of triple therapy with PPI (rabeprazole 10 mg or esomeprazole 20 mg twice daily), clarithromycin 200 mg twice daily, and amoxicillin 750 mg twice daily; 18 patients showed successful eradication. Second-line eradication (PPI, metronidazole 250 mg twice daily, and amoxicillin 750 mg twice daily for 1 week) was performed in 15 patients for whom the first-line eradication treatment was unsuccessful. *H. pylori* was not eradicated in one patient following second-line eradication; the individual received successful third-line eradication (PPI, 750 mg amoxicillin twice daily, and 100 mg sitafloxacin twice daily for 1 week). In cases of eradication failure, second-and third-line eradication were performed without assessing symptoms. However, regarding PPI usage, administration was limited to the eradication period, with discontinuation of PPIs for over a month after 1 week of eradication treatment. Symptom assessment took place after this discontinuation period. Patients whose dyspepsia and total scores decreased to ≤50% after *H. pylori* eradication were defined as Responders, whereas the other patients were defined as Non-responders [29].

A comparative analysis of various factors such as sex, age, observation period, BMI, smoking habits, and serum PG levels was performed between the two groups. ROC curves were constructed to extract the corresponding cut-off values that were used to determine sensitivity and specificity.

### 2.4. Statistical Analysis

Sample size was calculated using a two-sided alpha level of 0.05 and a power of 80%. The required sample size was calculated to be 28 cases based on the predicted values derived from previous research data [30]. In this study, there were 14 Responders and 19 Non-responders, thus meeting the required sample size.

The EZR software version 1.40 was used for data management and analysis. Continuous variables are presented as means ± standard deviations. Student’s *t*-test was used to compare factors affecting Responders and Non-responders. The symptom scores among the groups were compared using the Wilcoxon’s signed-rank test. Categorical variables were compared using the chi-square and Fisher’s exact tests. Multivariate logistic regression analysis was performed with BMI, PG I/II ratio, and smoking habits as independent variables and eradication efficacy as the dependent variable. The adjusted OR was calculated using multiple logistic regression analyses. All *p* values were two tailed, and statistical significance was considered at *p* value < 0.05.

## 3. Results

### 3.1. Characteristics of Responders and Non-Responders

The Responder and Non-responder groups had 14 and 19 patients, respectively (Tables S1 and S2). Regarding the symptoms of Responders, in 13 of 14 cases, dyspepsia scores improved after *H. pylori* eradication, as did reflux scores (except for one case where the reflux score increased from 2 to 3; Table S1). Table 1 shows the sex, age, observation period, BMI, smoking habit, and serum PG levels of the participants in both groups. Mean age, observation period, and BMI did not differ significantly between the groups. The univariate analysis revealed that sex, age (<70 vs. ≥70 years), BMI (<25 vs. ≥25 kg/m^2^), smoking habits (non-smoker vs. past and current smokers), alcohol consumption (nondrinkers vs. past and current drinkers), and the presence or absence of underlying diseases did not differ significantly between the groups. The serum PG I level was 62.8 ± 24.3 ng/mL in Responders, which was higher than that in Non-responders (55.3 ± 29.0 ng/mL). In contrast, the serum PG II level in Responders (19.8 ± 8.1 ng/mL) was lower than that in Non-responders (23.5 ± 9.1 ng/mL). As a result, a significant difference in the PG I/II ratio was observed between Responders (3.4 ± 1.2) and Non-responders (2.3 ± 1.0) (*p* = 0.006).

### 3.2. Cut-off PG I/II Ratio to Predict Sustained Dyspeptic Symptoms

**Figure 2:**
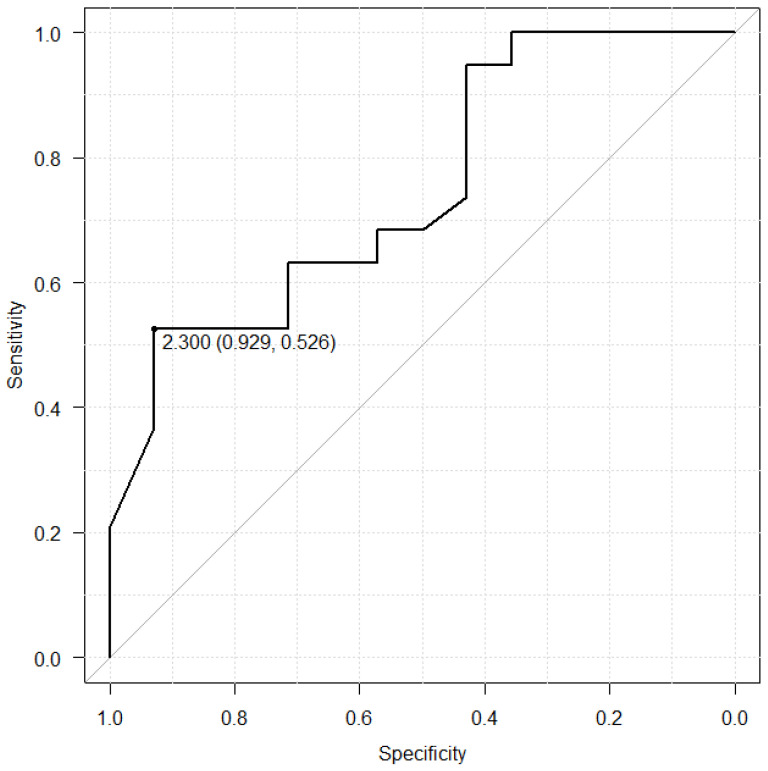
Cut-off value of pepsinogen (PG) I/II to predict sustained dyspeptic symptoms following *Helicobacter pylori* eradication.

Figure 2 shows the receiver operating characteristic (ROC) curve obtained from the 33 patients for predicting sustained dyspeptic symptoms. The area under the curve of the ROC analysis was 0.748 (95% CI: 0.578–0.918) for PG I/II, and the optimal PG I/II cut-off value was 2.3, with a sensitivity of 0.929, specificity of 0.526, positive predictive value of 0.591, and negative predictive value of 0.909.

### 3.3. Changes in Modified FSSG Scores Regarding PG I/II

Table 2 shows the modified FSSG score changes in the two groups classified by a cut-off value of 2.3 for the PG I/II ratio. After successful eradication, all scores significantly decreased in the group with a PG I/II ratio > 2.3. In contrast, a significant reduction was observed only in the dyspepsia scores in the group with a PG I/II ratio ≤ 2.3. However, the reduction was small (from 9 to 7), and the dyspeptic symptoms persisted. No significant decrease was observed in the total or reflux scores.

### 3.4. Multivariate Logistic Regression Analysis of Predictive Factors Associated with No Amelioration of Dyspeptic Symptoms after H. pylori Eradication

Multivariate logistic regression analysis was performed using BMI, PG I/II ratio, smoking habits, alcohol consumption, and underlying diseases as independent variables and eradication efficacy as the dependent variable. The adjusted odds ratio (OR) for non-responders was 20.1 (95% CI: 1.2–316.0, *p* = 0.033) for a PG I/II ratio ≤ 2.3 and 7.8 (95% CI: 1.1–55.9, *p* = 0.041) for smoking habits (Table 3). In contrast, no significant association with BMI, alcohol consumption, and underlying diseases was observed (BMI: 95% CI: 0.5–24.4, *p* = 0.215, alcohol consumption habits: 95% CI: 0.1–5.6, *p* = 0.839, and underlying diseases: 95% CI: 0.2–12.8, *p* = 0.652).

## 4. Discussion

In this study, we found that patients whose dyspeptic symptoms were ameliorated after *H. pylori* eradication had higher serum PG I levels, lower serum PG II levels, and significantly higher PG I/II ratios than those whose dyspepsia symptoms persisted.

Serum PG levels reflect gastric mucosal inflammation and glandular atrophy extent [31]. PG I is produced by the chief and mucous neck cells of the fundic glands. The serum PG I level is positively correlated with gastric acid secretion [22]. PG II is produced by the chief and mucous neck cells, as well as by the cells in the pyloric glands. The serum PG II level reflects the degree of gastric mucosal inflammation. When *H. pylori* infects the gastric mucosa, serum PG I and PG II levels increase. In our study, the increase in PG II level was more apparent than that of PG I, and the PG I/II ratio decreased (Figure S1). Along with the extent of glandular atrophy in the stomach corpus, serum PG I levels gradually decreased, whereas the extent of decrease in the PG II levels was relatively low. Consequently, the PG I/II ratio is correlated with the progression of glandular atrophy. The serum PG I level and PG I/II ratio have been used as glandular atrophy markers in gastric cancer screening using the serum PG test method [32,33,34].

In this study, serum PG I levels in Responders were higher than those in Non-responders. As PG I levels indicate the acid secretory potential of the stomach, an increase in the PG I level might indicate mild gastric mucosal atrophy and relatively preserved parietal cells. In contrast, serum PG II levels in Responders were relatively low, suggesting that gastric mucosal inflammation was mild. The PG I/II ratio was significantly higher in Responders than in Non-responders. Therefore, the extent of gastric mucosal atrophy was lower in Responders than in Non-responders. Additionally, the dyspepsia scores of the modified FSSG questionnaire after successful eradication improved significantly in the group with a PG I/II ratio > 2.3. Therefore, the PG I/II ratio cut-off value can be used to predict whether dyspeptic symptoms are likely to be alleviated after *H. pylori* eradication. In the multivariate logistic regression analysis, a low PG I/II ratio (PG I/II ratio ≤ 2.3) and smoking habits were associated with the ineffectiveness of *H. pylori* eradication treatment on dyspeptic symptoms.

Several studies have been conducted on PG and *H. pylori*-associated dyspepsia in Japanese patients. Tahara et al. studied serum PG levels in patients infected with *H. pylori*, where 38 patients had dyspeptic symptoms and 21 had no dyspeptic symptoms. These studies revealed that serum PG I levels were higher in patients with dyspeptic symptoms than in those without dyspeptic symptoms [35]. Kawamura et al. administered *H. pylori* eradication treatment to 45 patients with dyspeptic symptoms and evaluated their symptoms after 12 months [30]. After successful eradication, 34 patients had *H. pylori*-associated dyspepsia and 11 had FD. Moreover, dyspeptic symptoms were abated in 76% of the patients. The serum PG I levels before *H. pylori* eradication were significantly lower in patients with *H. pylori*-associated dyspepsia than in those with FD. However, the PG I/II ratio did not differ significantly between the two groups. A previous multivariate logistic regression analysis revealed that low serum PG II level is a useful predictor of *H. pylori*-associated dyspepsia [30]; however, the serum PG I level and PG I/II ratio were inconsistent with our results. In contrast, regarding the NNT for dyspeptic symptoms with *H. pylori* eradication, in addition to the previously mentioned report [15], a worldwide meta-analysis reported a value of 14 [36]. Similarly, in an Asian meta-analysis, the NNT was 15 [37]. Based on these data, symptomatic improvement after *H. pylori* eradication would be achieved in only 7% of patients. Our data showed that 42% (14/33) of the patients responded to eradication. Such a difference could be caused by the difference in the method of evaluating symptoms, including in the placebo response group. We used the modified FSSG questionnaire, whereas Kawamura et al. diagnosed FD based on two or more points on a five-point Likert scale of gastrointestinal symptoms [30]. Therefore, patients with relatively mild symptoms were diagnosed with FD, and the efficacy of *H. pylori* eradication may have been overestimated. In addition, the evaluation methods for dyspeptic symptoms and FD vary even in the aforementioned meta-analysis, thus necessitating future standardization of the evaluation methods for FD and further investigations into the matter.

The PG I/II ratio is correlated with the grade of glandular atrophy and gastric emptying [17]. Delayed gastric emptying is thought to cause dyspeptic symptoms. In this study, gastric mucosal atrophy was less progressive in Responders than in Non-responders. In patients with a PG I/II ratio ≤ 2.3, dyspeptic symptoms were not ameliorated by *H. pylori* eradication. In patients with severe gastric glandular atrophy, the number of parietal cells that secrete acids was reduced. The recovery of gastric acid secretion after *H. pylori* eradication is expected to be significant in patients with less glandular atrophy in the stomach corpus [38,39]. Consequently, the amelioration of delayed gastric emptying may contribute to the alleviation of dyspeptic symptoms. In contrast, some previous reports concluded that *H. pylori* eradication does not alter gastric emptying. Koskenpato et al. reported that in *H. pylori*-positive patients with dyspeptic symptoms, the eradication of *H. pylori* did not impact gastric emptying when measured using parameters such as postlag 50% retention time for solids (T50), gastric emptying half-time for liquids (T1/2), solid lag duration, and intragastric distribution of solids [34]. They concluded that while the eradication of *H. pylori* did not affect gastric emptying, the long-term trend in individual gastric emptying rates remained stable [40]. However, like the case for this study, these previous studies did not distinguish between patients with FD and *H. pylori*-associated dyspepsia. Some FD patients in those studies might have had *H. pylori*-associated dyspepsia. Therefore, the conclusions in those studies might differ from the findings in this research. Further investigation is needed to differentiate and examine the numbers of patients with FD and those with *H. pylori*-associated dyspepsia in future studies.

Furthermore, in the present study, patients with a smoking habit also sustained dyspeptic symptoms after *H. pylori* eradication. Smoking habits are associated with dyspeptic symptoms [41], and some reports have shown that past and current smoking habits are among the causes of these symptoms [42]. The association between smoking and dyspeptic symptoms can be explained by the effects of smoking on gastric function. Smoking affects gastric physiology, particularly by delaying gastric emptying [43,44], resulting in dyspeptic symptoms. Additionally, smoking is associated with changes in duodenal mucosal permeability and eosinophilic infiltration and contributes to abnormal gastric motility and increased gastric irritability in patients diagnosed with FD [42,43,44,45,46,47,48,49,50,51,52]. Duodenal eosinophilia is also correlated with smoking habits [53]. The interaction among smoking, duodenal mucosal changes, and gastrointestinal motility underscores the multifaceted nature of dyspeptic symptoms in patients with a smoking habit.

Previous studies have indicated that the intensity of dyspeptic symptoms increased with BMI [54] and is significantly higher at a BMI ≥ 25 kg/m^2^ [26]. Some studies have suggested a certain degree of association between alcohol consumption and FD [55,56,57]; however, several other studies have indicated that this association is weaker for alcohol consumption than for other factors [58,59,60]. In the present study, we observed no difference in the BMI and alcohol consumption habits between the two groups. Therefore, our results indicate that BMI and alcohol consumption habits do not adequately predict dyspeptic symptom resolution following *H. pylori* eradication. Among underlying diseases, hypertension, hyperlipidemia, and diabetes mellitus can contribute to the occurrence of FD [55,56]. However, research provides inconsistent and unclear evidence regarding their relationship and mechanisms. In the present study, we found no association between underlying diseases and dyspeptic symptoms. Nonetheless, this lack of association could be attributed to the failure to individually examine each underlying disease.

This study had some limitations. First, the sample size was small. The number of patients with *H. pylori* infection showing dyspeptic symptoms was limited among the patients enrolled in this multicenter study, which was also influenced by our use of the modified FSSG questionnaire with cut-off values of high sensitivity and specificity in the rigorous assessment of dyspeptic symptoms. The sensitivity of the questionnaire scoring system could affect the diagnostic rate and reported incidence rate of *H. pylori*-associated dyspepsia, and thus, more attention should be paid to this aspect. Furthermore, due to the small sample size, we were unable to conduct a multivariate analysis incorporating additional potentially relevant factors, such as food ingestion (salty, spicy, and meat) and socioeconomic status. Second, the observation period was short. To diagnose *H. pylori*-associated dyspepsia, follow-up for more than 6 months after successful *H. pylori* eradication is necessary. However, in this study, we assessed dyspeptic symptoms during the verification of *H. pylori* eradication approximately 3 months after eradication treatment. Clinically, patients whose dyspeptic symptoms were sustained after *H. pylori* eradication were often treated with acid secretion inhibitors and prokinetics. Long-term follow-up was difficult because the patients had been administered drugs that affected serum PG levels within 6 months after eradication. Therefore, we evaluated the improvement in dyspeptic symptoms rather than diagnosing FD. Third, the association of a low PG I/II ratio with unlikely symptom improvement suggests that these patients might have atrophic gastritis; therefore, their symptoms might be related to insufficient acid secretion rather than *H. pylori* infection. However, endoscopy with biopsy was not performed to confirm this hypothesis, presenting a significant limitation. However, determining factors related to *H. pylori*-associated dyspepsia that have not been clearly identified is important, and this study appears to be of clinical significance.

## 5. Conclusions

The PG I/II ratio and smoking habits are associated with the effects of *H. pylori* eradication on dyspeptic symptoms. The PG I/II ratio cut-off value can be used to identify patients who are likely to experience the resolution of dyspeptic symptoms following *H. pylori* eradication. Patients with a PG I/II ratio ≤ 2.3 and a history of smoking are likely to experience sustained symptoms, even after *H. pylori* eradication, resulting in the diagnosis of FD. In these patients, early treatment with acid secretion inhibitors and prokinetics should be considered (without waiting for 6 months) if dyspeptic symptoms are sustained after *H. pylori* eradication.

## Figures and Tables

**Table 1:** Characteristics of Responders and Non-responders.

Variables		Responders (n = 14)	Non-Responders (n = 19)	*p*-Value
Sex (%)	Male	4 (28.6)	8 (42.1)	0.486
	Female	10 (71.4)	11 (57.9)	
Age (years)		52.1 ± 13.6	57.0 ± 12.0	0.279
Age (%)	<70	12 (85.7)	17 (89.5)	1
	≥70	2 (14.3)	2 (10.5)	
Observation period (month)		3.3 ± 2.1	2.8 ± 1.2	0.391
BMI (kg/m^2^)		23.0 ± 4.1	23.7 ± 4.1	0.658
BMI (%)	<25	10 (71.4)	9 (47.4)	0.286
	≥25	4 (28.6)	10 (52.6)	
Smoking (%)	Non-smoker	11 (78.6)	8 (42.1)	0.073
	Past/Current smoker	3 (21.4)	11 (57.9)	
Alcohol consumption (%)	Non-drinker	7 (50.0)	8 (42.1)	0.733
	Past/Current drinker	7 (50.0)	11 (57.9)	
Underlying diseases (%)	Absence	9 (64.3)	6 (31.6)	0.085
	Presence	5 (35.7)	13 (68.4)	
PG I (ng/mL)		62.8 ± 24.3	55.3 ± 29.0	0.437
PG II (ng/mL)		19.8 ± 8.1	23.5 ± 9.1	0.229
PG I/II ratio		3.4 ± 1.2	2.3 ± 1.0	0.006

Abbreviations: BMI, body mass index; PG, pepsinogen.

**Table 2:** Changes in revised Frequency Scale for the Symptoms of Gastroesophageal Reflux Disease (FSSG) scores before and after *Helicobacter pylori* eradication.

Variables		PG I/II Ratio (Median)	
		>2.3 (n = 22)	≤2.3 (n = 11)
Total scores	Pre	15	14
	Post	4.5 **	14
Dyspepsia scores	Pre	9.5	9
	Post	2.5 **	7 *
Reflux scores	Pre	5	5
	Post	2 **	5

Data are presented as the median (range); * *p* < 0.05 and ** *p* < 0.01 vs. before *H. pylori* eradication.

**Table 3:** Multivariate logistic regression analysis of predictive factors for persistent dyspepsia after *H. pylori* eradication.

Variables		Adjusted OR	95% CI	*p*-Value
BMI (kg/m^2^)	<25.0	1		
	≥25.0	3.5	(0.5–24.4)	0.215
PG I/II ratio	>2.3	1		
	≤2.3	20.1	(1.2–316.0)	0.033
Smoking	Non-smoker	1		
	Past/Current smoker	7.8	(1.1–55.9)	0.041
Alcohol consumption	Non-drinker	1		
	Past/Current drinker	0.8	(0.1–5.6)	0.839
Underlying diseases	Absence	1		
	Presence	1.6	(0.2–12.8)	0.652

CI, confidence interval; OR, odds ratio.

## Data Availability

The data that support the findings of this study are available from the first author, K.Y., upon reasonable request.

## Supplementary Materials

The following supporting information can be downloaded at: https://www.mdpi.com/article/10.3390/life14080935/s1, Table S1: Profile of all patients in the Responder group; Table S2. Profile of all patients in the Non-responder group; Table S3. Modified Frequency Scale for the Symptoms of Gastroesophageal Reflux Disease, Figure S1: Location of pepsinogen I and II secretion.
