# Supplementary material for: Factors Predicting Effectiveness of Eradication Therapy for Helicobacter pylori-Associated Dyspepsia Symptoms
Source: Life (Basel). 2024 Jul 25;14(8):935. doi: 10.3390/life14080935 (PMC11355128; doi:10.3390/life14080935)
Supplement: Supplementary file 1 [file life-14-00935-s001.zip › Figure S1 .pptx]

## Slide 1
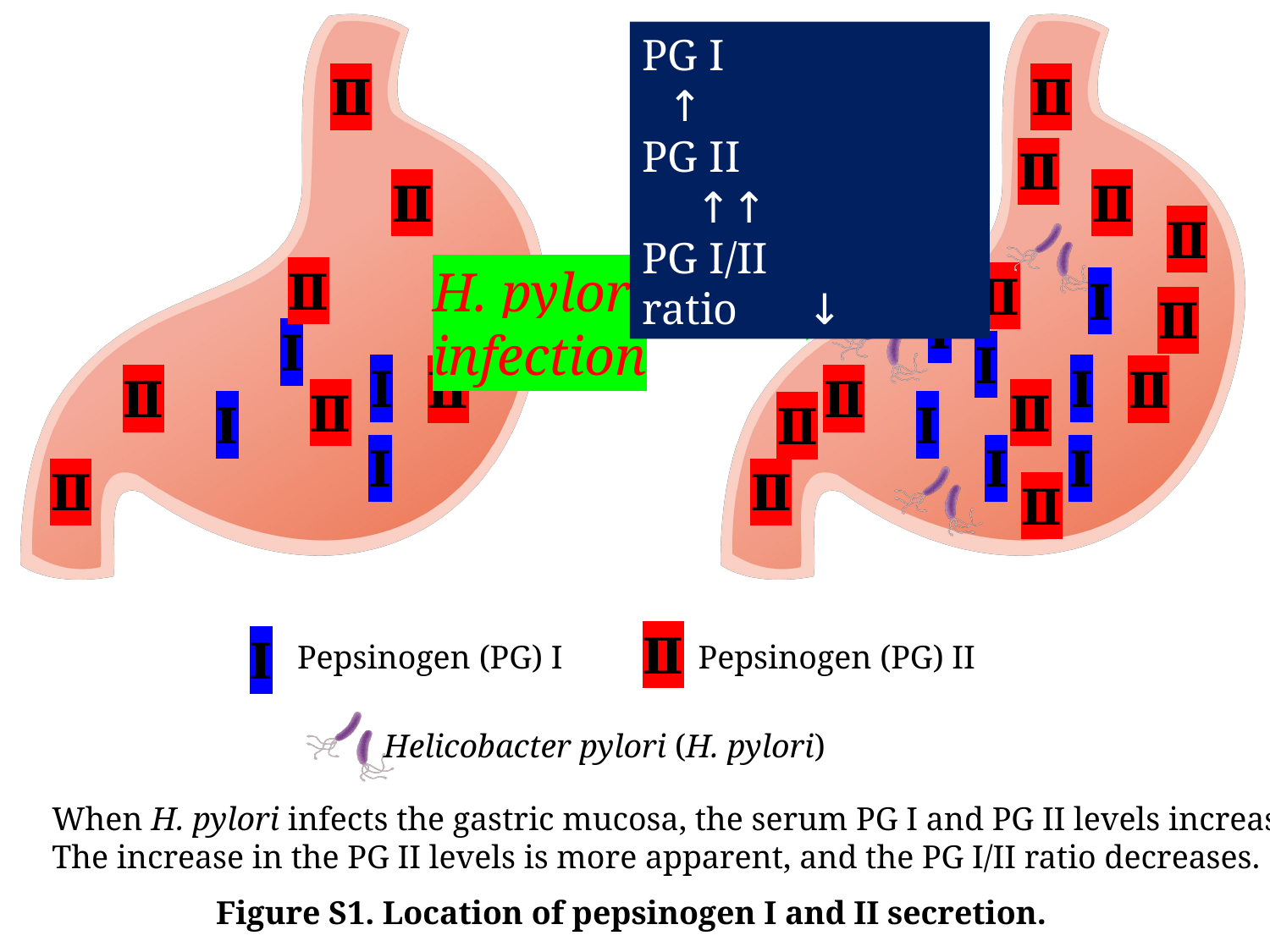

PG I　 　　　　　　　　　 　 　 ↑
PG II　　　 　 　 ↑↑
PG I/II　ratio　 ↓
Ⅱ
Ⅱ
Ⅱ
Ⅱ
Ⅱ
Ⅱ
H. pylori infection
Ⅱ
Ⅱ
Ⅰ
Ⅱ
Ⅰ
Ⅰ
Ⅰ
Ⅰ
Ⅰ
Ⅱ
Ⅱ
Ⅱ
Ⅱ
Ⅱ
Ⅱ
Ⅰ
Ⅰ
Ⅱ
Ⅰ
Ⅰ
Ⅰ
Ⅱ
Ⅱ
Ⅱ
Ⅱ
Ⅰ
Pepsinogen (PG) I
Pepsinogen (PG) II
Helicobacter pylori (H. pylori)
When H. pylori infects the gastric mucosa, the serum PG I and PG II levels increase.
The increase in the PG II levels is more apparent, and the PG I/II ratio decreases.
Figure S1. Location of pepsinogen I and II secretion.
