# Supplementary material for: Factors Predicting Effectiveness of Eradication Therapy for Helicobacter pylori-Associated Dyspepsia Symptoms
Source: Life (Basel). 2024 Jul 25;14(8):935. doi: 10.3390/life14080935 (PMC11355128; doi:10.3390/life14080935)
Supplement: Supplementary file 1 [file life-14-00935-s001.zip › Supplementary Tables S1-S3.pdf]

Table S1. Profile of all patients in Responder group.

	Age (years)	Sex	Observation period (months)	BMI (kg/m²)	Smoking	Alcohol consumption	Underlying diseases	PG I (ng/mL)	PG II (ng/mL)	PG I/II ratio		Dyspepsia scores		Total scores	
												Pre	Post	Pre	Post
	49	F	2.7	20.4	−	−	−	64.9	13.9	4.7		11	1	17	1
	36	F	5.3	18.2	−	+	−	57.7	24.3	2.4		8	0	12	0
	61	F	1.9	20.3	−	+	−	75.4	31.3	2.4		7	0	9	3
	28	F	1.9	23.5	−	−	−	50.5	18.6	2.7		12	1	15	3
	70	F	1.9	26.5	−	−	HT	37.3	8.3	4.5		8	2	15	4
	37	F	1.9	17.2	−	−	−	56.4	18.3	3.1		13	0	21	0
	45	M	1.9	26.5	−	−	−	78.8	15.4	5.1		8	3	13	6
	54	M	2.1	26.1	−	+	HT	59.5	14.4	4.1		7	1	12	2
	53	F	8.5	19.8	+	−	−	55.8	22.8	2.4		12	0	19	0
	40	F	6.2	33.3	−	+	Asthma	41.8	16.2	2.6		11	0	19	2
	66	M	4.7	23.1	−	+	−	46.7	8.6	5.4		12	5	12	5
	63	M	1.7	22	+	+	Gout	113.1	31.3	3.6		8	3	14	4
	72	F	3.8	21.8	+	−	−	32.4	19.3	1.7		15	0	16	0
	55	F	2.4	23.9	−	+	HL, DM	109.4	34.1	3.2		10	2	16	4
Mean	52.1		3.3	23				62.8	19.8	3.4	Median	10.5	1	15	2.5
SD	13.6		2.1	4.1				24.3	8.1	1.2	IQR	(8–12)	(0–2)	(12–17.5)	(0–4)

Abbreviations: BMI, body mass index; IQR, interquartile range; PG, pepsinogen; SD, standard deviation; M, male; F, female; HT, hypertension; HL, hyperlipidemia.

Table S2. Profile of all patients in Non-responder group.

	Age (years)	Sex	Observation period (months)	BMI (kg/m²)	Smoking	Alcohol consumption	Underlying diseases	PG I (ng/mL)	PG II (ng/mL)	PG I/II ratio		Dyspepsia scores		Total scores	
												Pre	Post	Pre	Post
	47	F	1.8	19.3	+	−	Basedow's disease, HL	86.3	24.5	3.5		16	15	17	15
	65	F	1.5	19.2	−	−	Iron-deficiency anemia	9.9	12.9	0.8		10	11	16	23
	60	M	2.6	18.6	+	+	HT, HL	40	27.7	1.4		8	7	14	10
	61	F	5.5	25.5	−	−	−	88.6	26.5	3.3		7	5	10	10
	65	F	4.2	28	−	+	HT, HL	46.4	27.3	1.7		15	10	19	30
	58	F	2.2	19.5	−	+	HT	73.6	44.3	1.7		8	4	12	8
	63	M	4.8	21	+	+	HT	108.7	34.3	3.2		9	2	15	8
	38	M	2.1	29.8	+	+	−	54	13.8	3.9		10	7	14	10
	29	F	1.7	17.8	+	+	−	51.4	20.7	2.5		7	9	9	12
	66	M	3.2	28.2	+	−	HT	59.4	16.9	3.5		8	5	11	5
	52	F	3.7	24	−	−	−	65.9	26	2.5		14	8	15	8
	74	M	2	28.3	+	+	HT, DM	25.2	13.7	1.8		7	5	8	5
	50	F	3	25.2	+	+	−	93.9	40.4	2.3		16	11	28	16
	71	M	1.9	21.6	−	+	Allergic rhinitis	12.4	16.5	0.8		9	6	14	10
	60	F	2.2	27.5	−	−	HT	25.4	20.4	1.2		9	9	9	14
	64	M	3	27.3	+	+	HT, Cerebral infarction	55.5	26.3	2.1		8	6	13	15
	53	F	2.1	18.2	−	−	Insomnia	25.2	14.6	1.7		9	8	15	16
	39	F	2.2	25.8	+	−	−	88.2	26	3.4		11	6	18	9
	68	M	4.5	25.3	+	+	HT, arrhythmia, Gout	41	14.1	2.9		9	10	15	12
Mean	57		2.8	23.7				55.3	23.5	2.3	Median	9	7	14	10
SD	12		1.2	4.1				29	9.1	1.0	IQR	(8–11)	(5–10)	(11–16)	(8–15)

Abbreviations: BMI, body mass index; IQR, interquartile range; PG, pepsinogen; SD, standard deviation; M, male; F, female; HT, hypertension; HL, hyperlipidemia; DM, diabetes mellitus.

Table S3. Modified Frequency Scale for the Symptoms of Gastroesophageal Reflux Disease Questionnaire

Question		Never	Occasionally (1–2 days per week)	Sometimes (3–4 days per week)	Often (5–6 days per week)	Always
1	Do you get heartburn?	0	1	2	3	4
2	Does your stomach get bloated?	0	1	2	3	4
3	Does your stomach ever feel heavy after meals?	0	1	2	3	4
4	Do you sometimes subconsciously rub your chest with your hand?	0	1	2	3	4
5	Do you ever feel sick after meals?	0	1	2	3	4
6	Do you get heartburn after meals?	0	1	2	3	4
7	Do you have an unusual (e.g. burning) sensation in your throat?	0	1	2	3	4
8	Do you feel full while eating meals?	0	1	2	3	4
9	Do some things get stuck when you swallow?	0	1	2	3	4
10	Do you get bitter liquid (acid) coming up into your throat?	0	1	2	3	4
11	Do you burp a lot?	0	1	2	3	4
12	Do you get heartburn if you bend over?	0	1	2	3	4
13	Do you get epigastric pain (burning) after meals?	0	1	2	3	4
14	Do you get epigastric pain (burning) before meals?	0	1	2	3	4

We referred to seven questions (1, 4, 6, 7, 9, 10, and 12) as the reflux symptom questions, and these were used to calculate the reflux score. The other seven questions (2, 3, 5, 8, 11, 13, and 14) were used to calculate the dyspepsia score.
